# An Intestinal Microbiome Intervention Affects Biochemical Disease Activity in Patients with Antiphospholipid Syndrome

**DOI:** 10.1055/s-0044-1788653

**Published:** 2024-08-05

**Authors:** Valérie L. B. I. Jansen, Dagmar J. M. van Mourik, Mark Davids, Kika van Bergen en Henegouwen, Tessa Noordermeer, Johannes H. M. Levels, Maarten Limper, Michiel Coppens, Max Nieuwdorp, Rolf T. Urbanus, Saskia Middeldorp, Thijs E. van Mens

**Affiliations:** 1 Department of Vascular Medicine, Amsterdam UMC Location AMC, University of Amsterdam, Amsterdam, The Netherlands; 2 Amsterdam Cardiovascular Sciences, Pulmonary Hypertension and Thrombosis, Amsterdam, The Netherlands; 3 Amsterdam Reproduction and Development Research Institute, Amsterdam, The Netherlands; 4 Department of Experimental Vascular Medicine, Amsterdam UMC location AMC, University of Amsterdam, Amsterdam, The Netherlands; 5 Department of Medicine - Thrombosis and Haemostasis, Leiden University Medical Center, Leiden, The Netherlands; 6 Center for Benign Haematology, Thrombosis and Haemostasis, Van Creveldkliniek, University Medical Center Utrecht, Utrecht, The Netherlands; 7 Department of Rheumatology and Clinical Immunology, University Medical Center Utrecht, Utrecht, The Netherlands; 8 Department of Internal Medicine and Radboud Institute of Health Sciences (RIHS), Radboud University Medical Center, Nijmegen, The Netherlands

**Keywords:** antiphospholipid syndrome, autoimmune disease, intestinal microbiome, gut microbiome

## Abstract

**Background** The origin of autoantibodies in patients with antiphospholipid syndrome (APS) is unknown. The gut microbiome contributes to autoimmunity and contains peptide homologues to the main APS autoantigen, which affect disease activity in animal models. Alteration of the gut microbiota with vancomycin diminishes disease activity in mice but no data on the effect of gut microbiota alteration in APS patients are available to date.

**Objective** To evaluate whether the gut microbiome affects disease activity in human APS.

**Methods** This was a pre–post design intervention study in APS patients with stable disease and no gastrointestinal comorbidity. Subjects received oral vancomycin, 500 mg four times daily for 7 days, previously shown to alter gut microbiota composition without systemic effects. Disease activity was assessed at four time points by measuring a panel of clinical phenotype-related biomarkers: antiphospholipid antibodies (APLAs), complement and inflammation markers, and hemostatic parameters. The primary outcome was the composite of the biomarker panel determined by multilevel principal component analysis.

**Results** A total of 15 subjects completed the study. The primary outcome, the first principal component of the biomarker panel data, was significantly different after 7 days of vancomycin treatment ( *p* = 0.03), but not at day 42. APLA titers were unaffected. Unexpectedly, 4 out of 15 patients were negative for APLAs at baseline. In a post-hoc analysis, there was a prolonged effect for subjects with positive antibodies at baseline ( *p* = 0.03). In subjects with negative APLAs at baseline, the intervention showed no effect.

**Conclusion** The intestinal microbiome affects the biochemical disease activity in APS patients. The mechanism is yet unknown but appears to be APS-specific.

## Introduction

Antiphospholipid syndrome (APS) is a prothrombotic autoimmune disorder that is characterized by obstetric or thrombotic morbidity in the persistent presence of antiphospholipid antibodies (APLAs). 1 The main autoantigen these antibodies are directed against is circulating plasma protein β-2-glycoprotein 1 (β2GP-1). The etiology of APLAs remains elusive. Consequently, no curative treatment is available and patients are treated with anticoagulants, a treatment with both suboptimal safety in terms of bleeding and suboptimal efficacy, with recurrent thrombosis and obstetric complications occurring under treatment.

The APS-defining antibodies are characterized by persistence over time, but transient APLAs are relatively common in individuals without APS. These transient antibodies are often triggered by infections. 2 The intestinal microbiome, the community of microorganisms that colonizes the intestinal tract, poses a chronic exposure to a broad variety of microbial antigens and is thus hypothesized to drive the formation of persistent APLAs. 3 The gut microbiota indeed can trigger autoimmunity through antigen cross-reactivity. For several autoimmune disorders, including APS, gut bacteria that contain peptide sequence homologues to the epitopes of autoantigens have been identified, resulting in antigen cross-reactivity. 4 5 6 For instance, the gut commensal *Roseburia intestinalis* contains peptide sequences homologous to the predominant B cell and T cell epitopes of β2GP-1. 5 Both APS patient plasma and a patient-derived monoclonal APLA indeed show cross-reactivity with this B cell epitope-mimicking bacterial protein. 5 Similarly, APS patient-derived T cells cross-react with the *R. intestinalis* T cell epitope homologue. Further experiments with (NZW x BXSB)F1 mice showed that immunization with and (immunization with and oral administration of) oral administration of the bacterium led to β2GP-1 cross-reactive antibodies and thrombosis, supporting a causal contribution of gut microbiota to murine APS. 5

The emerging evidence on the etiological role of the microbiome in autoimmunity provides new possible therapeutic targets. Treatment with oral vancomycin of APS (NZW x BXSB)F1 mice reduces anti-β2GP1 immunoglobulin G (IgG) titers, decreased cerebral, cardiac and pulmonary thrombi, and improves survival. 7 In systemic lupus erythematosus, a disease that strongly overlaps with APS, transfer of healthy microbiome to patients with therapy-unresponsive disease flares appears to diminish disease activity. 8

Both findings underline the therapeutic potential of microbiome-targeted therapies in autoimmune disease.

To this end, the current study aims to translate the above observations to human APS by determining whether the intestinal microbiota affect the biochemical disease activity in APS patients.

## Methods

### Study Population

We recruited patients with APS, whose diagnosis met the APS Sydney research criteria, from our tertiary Amsterdam University Medical Centers Vascular Medicine outpatient clinic and through advertisements with the Dutch APS patients association. 1 All subjects were included based on their historical APLA values and were not re-screened before start of the study. Positive APLAs were defined as positive lupus anticoagulant or anti-β2GP-1 IgG or IgM or anti-cardiolipin IgG or IgM titer above 40 GPL or MPL or above 99th percentile, on at least two occasions 12 weeks apart. Exclusion criteria were: age below 18 years, current use of antibiotics, current use of a vitamin K antagonist, history of gastroenteritis in the past month, history of inflammatory bowel disease, current pregnancy or pregnancy in the past 6 weeks, arterial or venous thrombosis in the past month, allergy to vancomycin or planned change in platelet aggregation inhibitors, anticoagulants or hormonal therapy during the study period. Subjects using vitamin K antagonist were excluded because of a potential effect of the intervention on the international normalized ratio.

The Medical Research Ethics Committee of the Amsterdam University Medical Centers approved the study. All participants provided written informed consent. The study was conducted in accordance with the Declaration of Helsinki. The study was registered in the Dutch trial register prior to initiation (NTR 7662).

### Study Design

All study participants received oral vancomycin 500 mg four times per day for 7 days. Vancomycin is a broad-spectrum antibiotic mainly effective against gram-positive bacteria that is poorly absorbed from the gut and has previously been shown to elicit a distinct shift in gut microbiota. 9 Blood, fecal and urine samples were taken at 7 days before start of treatment (day −7), at baseline (day 0), at end of treatment (day 7), and 6 weeks after start of treatment (day 42).

Venous blood was drawn with an 18G needle; all blood samples were processed within 3 hours after collection and stored at −80°C. Participants collected fecal samples at home within 24 hours preceding the study visit. Samples were stored at 4°C until study visit and then stored at −80°C until processing.

### Outcome

Given the incidence rates, clinical endpoints (i.e., thrombotic or obstetric events) were not a suitable outcome in this study design. Antibody profiles correlate with clinical outcomes but no single antibody test correlates strongly enough to function as a surrogate endpoint. Moreover, since the mechanism through which the intestinal microbiota influence APS remains elusive, it is unclear which are the optimal biomarkers to study such an effect. Therefore, we composed a comprehensive biomarker panel of parameters identified from the literature to be associated with the clinical APS phenotype ( Table 1 ). 1 10 11 12 13 14 15 16 17 18 The predefined primary outcome of the study was the first principal component of the biomarker panel data. This value collectively captures the maximized variance, while it is adjusted for inter-individual variance, in the combined clinical APS phenotype-related biomarkers and is thus considered to reflect the changes in disease activity.

**Table 1:** Biomarker panel

	Biomarker
Antiphospholipid antibodies	Lupus anticoagulant
	Anti-β2GP1 IgG, IgM, IgA
	Anti-cardiolipin IgG, IgM, IgA
	Anti-phosphatidylserine/prothrombin IgG, IgM
Primary hemostasis	Platelet count
	Light transmission aggregometry
	Platelet flow cytometry
	Von Willebrand factor
Secondary hemostasis	Activated partial thromboplastin time, prothrombin time
	Calibrated automated thrombogram
	Activated protein C resistance
NETosis	Citrullinated histone H3
Fibrinolysis	Clot lysis
	D-dimer
Complement	C3a
	C5a
Inflammatory markers	C-reactive protein
	Tumor necrosis factor α
	Interferon γ
	Interleukin-6

Abbreviation: IgA, immunoglobulin A; anti-?2GP1: Anti-β-2-glycoprotein-I.

### Biomarker Assays

In addition to APS criteria APLAs, we evaluated anti-cardiolipin IgA and anti-β2GPI IgA antibodies, because of their role in intestinal immunity, and we measured anti-phosphatidylserine/prothrombin antibodies. The APLA and lupus anticoagulant assays are described in detail in Supplementary Materials and Methods . Quantitative antibody titers and the numerical value of the normalized lupus anticoagulant ratio for both Russell's viper venom time and silica clotting time were included in the analysis.

Light transmission aggregometry was performed by inducing aggregation with three concentrations of adenosine diphosphate (ADP): 2, 5, and 10 μM. The following parameters of the aggregation curve were included in the analysis: primary slope, primary aggregation, maximal aggregation, final aggregation.

For platelet flow cytometry, platelets were either unstimulated or stimulated with a receptor-specific agonist either ADP, CRP-xl, PAR-1 AP, PAR-4 AP, or U46619. Platelets identified based on forward and side scatter were selected on GPIbα positivity or integrin αIIbβ3 positivity. Of the unstimulated integrin αIIbβ3-positive population, the percentages positive for glycoprotein VI and integrin α2β1 were included in the analysis. For the unstimulated and agonist-stimulated platelets, the percentages positive for anti-P-selectin and anti-fibrinogen of the GPIbα-positive population were included in the analysis.

For the calibrated automated thrombogram, thrombin generation curves were generated with 1 pM and respectively 5 pM of tissue factor. The following parameters were included in the analysis for both tissue factor concentrations: lag time, peak height, time to peak, velocity, and endogenous thrombin potential (ETP). Activated protein C (APC) resistance was measured by evaluating ETP in the presence of APC and 5 pM tissue factor. APC sensitivity ratios (ETP values measured in the presence of APC/ETP values in absence of APC) normalized to pool plasma were included in the analysis.

Clot lysis test was performed with and without carboxypeptidase inhibitor (CPI) to evaluate contribution of activation of thrombin activatable fibrinolysis inhibitor. Clotting time, clot lysis time without CPI, and clot lysis time with CPI were included in the analysis.

### Secondary Outcomes

We evaluated gut microbiota composition by 16S sequencing (Illumina), assessed intestinal permeability with lactulose mannitol test and plasma marker lipocalin-2 and measured fecal calprotectin and fecal short-chain fatty acids (SCFAs). 19 These assays are described in the Supplementary Materials and Methods section.

### Statistical Analysis

All analyses were performed in R statistics version 4.0.3. We performed multilevel principal component analysis using the mixOmics package. 20 21 Principal component analysis, a widely used form of machine learning, is a dimension reduction technique, used to enhance analysis and visualization of high dimensional data, i.e., high variable number datasets. It reduces the variables by capturing the maximum amount of variability in the data on new composite virtual variables, called principal components. Multilevel principal component analysis is a variant that is specifically applicable to repeated measurement data. This multilevel analysis deals with the high inter-individual variation relative to the intra-individual variation, which is the relevant parameter in this pre–post intervention design. For the principal component analysis, the biomarker panel data were centered and scaled. Nonnormally distributed data were transformed using log transformation. We replaced assay results below the lower limit of quantification with the lower limit divided by two. Variables with over 20% missing values were excluded from the analysis. The remaining missing data were imputed using the Non-linear Iterative Partial Least Squares (NIPALS) algorithm within the mixOmics package. Differences in the principal components, which followed a normal distribution, were tested using *t* -test comparing the averaged baseline values (time point day −7 and day 0 combined) to day 7 and to day 42.

Secondary outcomes (APLA titers, lipocalin-2, fecal SCFAs, and fecal calprotectin) were evaluated with paired *t* -test. Considering APLA profiles differ amongst APS patients, APLA titers were assessed as a composite value. For each subject we selected the APLAs that subject was positive for at baseline. These APLA values were normalized to the baseline value of that APLA in the individual subject. These normalized values were then averaged to create a composite APLA.

Microbiota data were analyzed using phyloseq, vegan, and mixOmics packages. Microbiota abundance was visualized using multi-level principal component analysis of the centered log-ratio transformed amplicon sequence variant counts.

## Results

A total of 16 APS patients were included in this study. One subject was excluded before start of treatment because of pregnancy. In total, 15 subjects completed the study, of which 14 were women. Baseline characteristics and APLA profiles of the participants who underwent treatment are depicted in Table 2 . Notably, 4 out of 15 study subjects tested negative for all APLA at baseline. Patients were included based on previous diagnostic APLA values and not rescreened before inclusion.

**Table 2:** Baseline characteristics

Characteristic	*N* = 15
Age, mean ± SD	39.3 ± 8.6
Female, *n* (%)	14 (93)
BMI, mean ± SD	26.6 ± 5.6
Ethnicity, *n* (%)	
Caucasian	13 (87)
Mixed	2 (13)
History of pregnancy morbidity, *n* (%)	
Fetal loss ≥ 10 weeks of gestation	8/12 (67)
> 2 early pregnancy losses	3/12 (25)
Preterm birth	2/12 (17)
History of thrombosis, *n* (%)	
Venous thrombosis	3 (20)
Arterial thrombosis	3 (20)
Concurrent autoimmunity, *n* (%)	
SLE	0 (0)
Other autoimmune disorder	2 (13)
Use of DOAC, *n* (%)	4 (27)
APLA positivity at diagnosis, *n* (%)	
Lupus anticoagulant	10 (67)
Anti-β-2-glycoprotein-I IgG	8 (53)
Anti-β-2-glycoprotein-I IgM	2 (13)
Anti-cardiolipin IgG	10 (67)
Anti-cardiolipin IgM	4 (27)
APLA positivity at baseline, *n* (%)	
Lupus anticoagulant	6 (40)
Anti-β-2-glycoprotein-I IgG	6 (40)
Anti-β-2-glycoprotein-I IgM	4 (27)
Anti-β-2-glycoprotein-I IgA	4 (27)
Anti-cardiolipin IgG	6 (40)
Anti-cardiolipin IgM	1 (6.7)
Anti-cardiolipin IgA	5 (33)
Anti-phosphatidylserine prothrombin IgG	6 (40)
Anti-phosphatidylserine prothrombin IgM	5 (33)
Triple positive (LAC, any aβ2GP1, any aCL)	3 (20)
No APLA	4 (27)

Abbreviations: APLA, antiphospholipid antibody; BMI, body mass index; DOAC, direct oral anticoagulant; IgA, immunoglobulin A; LAC, lupus anticoagulant; SD, standard deviation; SLE, systemic lupus erythematosus; aCL, anti-cardiolipin; a?2GP1, anti-β-2-glycoprotein-I.

### Gut Microbiota Composition

**Fig. 1:**
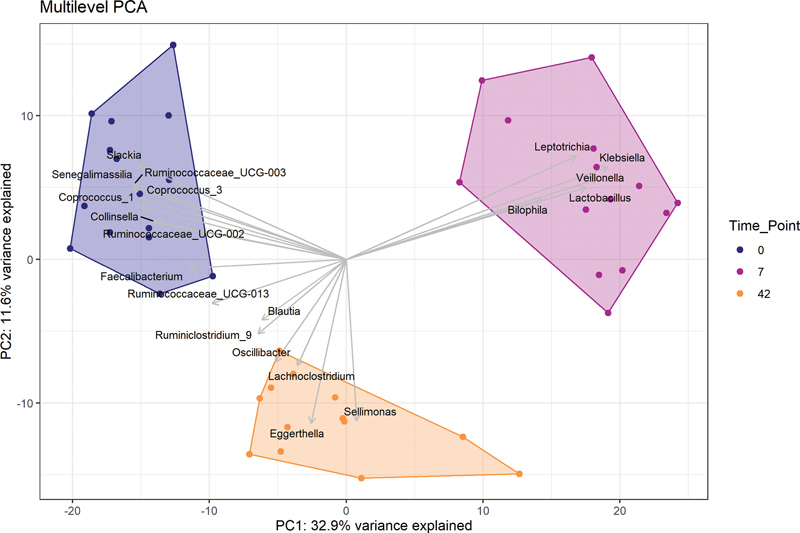
Multi-level PCA of the centered log-ratio transformed amplicon sequence variant counts. This provides a visual representation of the fecal microbiome abundance data at the three time points. Genera with the strongest ordination associations are indicated with arrows. PC, principal component; PCA, principal component analysis.

Treatment with vancomycin induced a distinct shift in gut microbiota composition directly after end of treatment which faded at 6 weeks after treatment ( Fig. 1 ). Fig. 1 shows the difference in the first two principal components (PC1 and PC2) of the abundance of the sequenced microbes between day 0 (blue), day 7 (purple), and day 42 (yellow).

### Primary Outcome

**Fig. 2:**
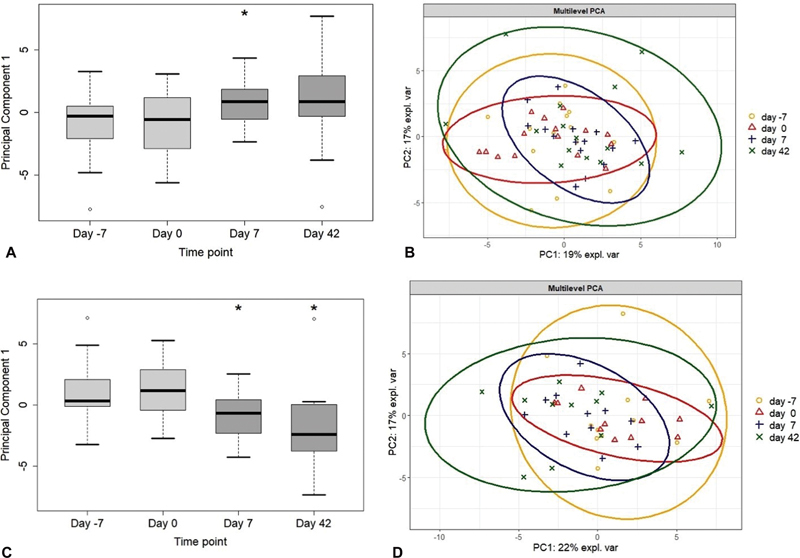
**Principal component analysis of the biomarker panel** . ( **A** ) Primary outcome principal component 1 differs between baseline and day 7 (absolute difference: 1.65, *p* = 0.03). ( **B** ) Multilevel PCA plot for all subjects at four time points. ( **C** ) Post-hoc analysis: principal component 1 for APLA-positive subjects ( *n* = 11) significantly differs between baseline and day 7 and between baseline and day 42 (absolute difference: −2.12, *p* = 0.02, and absolute difference: −3.00, *p* = 0.03, respectively). ( **D** ) Multilevel PCA plot for APLA-positive subjects at four time points. APLA, antiphospholipid antibody; PCA, principal component analysis; PC, principal component.

The primary outcome, the first principal component (PC1) of the biomarker panel, is depicted in Fig. 2A at the four time points. C-reactive protein, tumor necrosis factor-α, and interferon-γ were excluded from the analysis because the majority of measurements was below the limit of detection. PC1 differed between baseline and day 7 (absolute difference: 1.65, *p* = 0.03). There was no difference between baseline and day 42 (absolute difference: 1.89, *p* = 0.10).

A post-hoc analysis of the subgroup of patients with positive APLAs at baseline showed a significant difference in PC1 at both day 7 and day 42 compared with baseline ( Fig. 2C ) (absolute difference: −2.12, *p* = 0.02 and absolute difference: −3.00, *p* = 0.03, respectively). Of note, the reverse direction in absolute difference in the post-hoc analysis derives from the difference in principal coordinates in the subgroup data and does not signal an opposite biological effect.

**Fig. 3:**
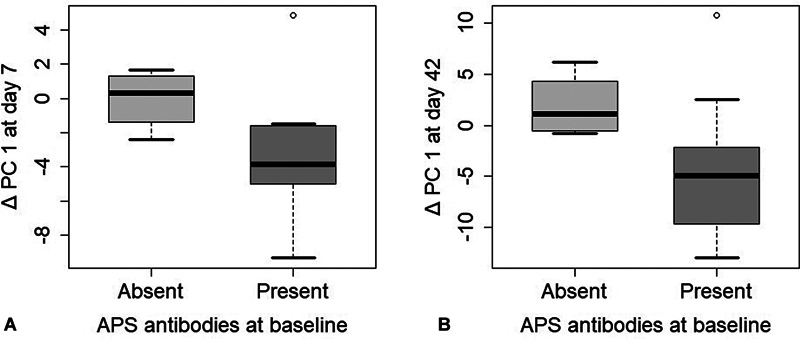
**Differences in the first principal component at two time points in patients with (*n* = 11) and without (*n* = 4) positive APLAs at baseline.** ( **A** ) Delta in PC1 at day 7 for patients with and without APLAs significantly differed ( *p* = 0.03). ( **B** ) Delta in PC1 at day 42 for patients with and without APLAs, significant difference ( *p* = 0.03). APLA, antiphospholipid antibody.

The finding of 4 out of 15 patients testing negative for all APLA was unanticipated. This did however create the possibility of investigating whether the effect on the main outcome (PC1) was APS-specific. We performed a post-hoc comparison between the subjects that had APLAs at baseline and those that tested negative for all antibodies. Delta values for PC1 were calculated by subtracting the PC1 value at day 7 and 42, respectively, from the combined preintervention values. The groups were compared on these delta-values using unpaired *t* -test. In the APLA positive group, the delta in PC1 was −3.44 at day 7, whereas in patients with negative APLAs, the PC1 delta was −0.03 ( *p* = 0.03) ( Fig. 3A ). A similar difference between these groups was found at day 42 with a PC1 delta of −4.46 in the patients with positive APLAs and 1.89 in subjects with absent APLAs at baseline ( *p* = 0.03) ( Fig. 3B ).

### Secondary Outcomes

#### Antiphospholipid Antibody Titers

**Fig. 4:**
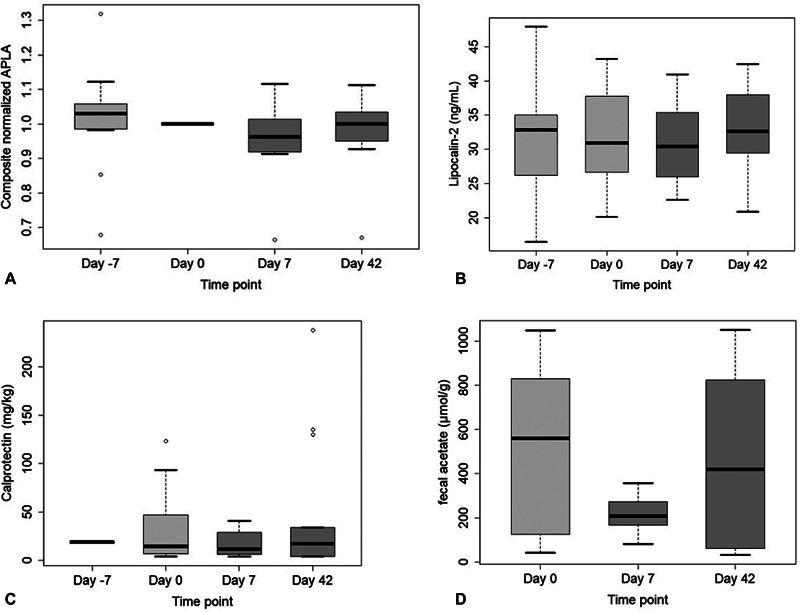
**Secondary outcomes at four time points.** ( **A** ) The normalized APLA composite for subjects with positive APLA at baseline ( *n* = 11). ( **B** ) Plasma lipocalin-2 levels for all subjects ( *n* = 15). ( **C** ) Fecal calprotectin levels for all subjects ( *n* = 15). ( **D** ) Fecal acetate levels for all subjects ( *n* = 15). APLA, antiphospholipid antibody.

Because APS patients have distinct APLA profiles, APLA levels were analyzed as a composite value reflecting the change in an individual's profile. This composite value did not differ between baseline and both day 7 and day 42 ( Fig. 4A ) (paired *t* -test, *p* = 0.25 and *p* = 0.56, respectively).

#### Intestinal Permeability

Neither plasma lipocalin-2 levels nor fecal calprotectin ( Fig. 4B, C ) levels showed a difference postintervention compared with baseline indicating no effect of vancomycin in gut permeability. All but one lactulose measurements were below the limit of detection, prohibiting comparison between the time points.

#### Fecal Short-Chain Fatty Acids

Of the fecal SCFAs, only acetate was different between day 0 and day 7 ( Fig. 4D , *p* = 0.04), but this was not statistically significant after Bonferroni correction for multiple testing.

## Discussion

The current study evaluated whether experimentally changing the intestinal microbiome affects the disease activity in human APS. The intervention of oral vancomycin, which is not absorbed from the gut, was effective in eliciting a substantial shift in the gut microbiota composition. This indeed resulted in a change in biochemical phenotype at the end of treatment, as collectively reflected by the panel of clinical APS phenotype-related biomarkers. The effect diminished after 6 weeks, but was prolonged in the subgroup with APS-defining antibodies present at baseline. The unanticipated subgroup without APLA at baseline showed no response to treatment in a post-hoc analysis. This suggests that the effect of this intervention is specific to APS patients. Overall, the data support a pathophysiological role of the microbiota in human APS.

The findings in APS patients complement in vitro data, mouse studies, and human observational studies on the role of the gut microbiota in APS. 3 5 7 Measuring disease activity poses a challenge in smaller human APS studies, given the infrequency of clinical events and the range of cell types and extracellular proteases involved in the pathogenesis. This novel approach represents an integral and unbiased method to assess the APS biochemical phenotype. The wide selection of biomarkers, which have been associated with the APS phenotype previously, ensures that the broad spectrum of APS pathophysiology is captured. The application of principal component analysis, with multilevel variant to account for the repeated measurement data structure, enables us to combine these data into a single representative outcome measure in an unbiased way.

The choice of experimental study design merits consideration, and was determined by the fact that APS is a rare disease. The prevalence of APS makes randomized studies, requiring a larger sample size, unfeasible for proof-of-concept studies such as performed here. The pre–post design remains valid for the primary objective of investigating any effect of the gut microbiome on disease biomarkers in APS patients. A pre–post design is sensitive to bias when a time-outcome effect is present. However, our inclusion criteria ensured patients in a stable disease state. For this chronic condition, variation within the short time span of the 7-week study duration is not to be expected. Moreover, as this was not a therapeutic study, randomization was not necessary to ensure comparability between exposure states (treated vs. untreated). Instead, patients functioned as their own controls maximizing statistical power to investigate our hypothesis in this relatively rare disease.

This study has some limitations. The use of the composite outcome hampers the interpretation of the effect size. The value is dimensionless and cannot be used to inform us on the clinical impact. A sample size calculation was not possible because the effect size could not be estimated with a dimensionless primary outcome. This methodological tradeoff was deemed necessary and functional, given that the aim was an assessment of whether, rather than how much, the microbiota affect APS in humans.

Another limitation is that four subjects tested negative for all APLAs. All subjects had a historic diagnosis of APS according to the Sydney criteria 1 but were not re-screened for APLAs directly before start of the study. Possible explanations for the discrepancy include seroconversion and interlaboratory variation, both of which are known to occur with APLAs. 22

We investigated several potentially involved mechanisms. The shift in disease activity is likely not to be mediated by a direct effect on APLA production or clearance as the intervention did not affect antibody titers. Previous work has shown an altered intestinal permeability in autoimmune disease in general and APS in particular. 5 23 However, we in fact found no evidence of increased gut permeability in our APS cohort. A modest effect too small to detect with the markers we used cannot be excluded. The lactulose mannitol test however, a gold standard for functional measurement of gut permeability, resulted in concentrations below the detection limit of our sensitive mass spectrometry assay for virtually all subjects. 19 24 However, since there is extensive interplay between the immune system and intestinal microbiota with an intact barrier function, increased intestinal permeability is not a prerequisite for an intestinal microbiota-mediated effect on disease activity.

A direct systemic effect of vancomycin on the biomarker panel is unlikely because of its poor intestinal absorption. Moreover, the effect appeared to be APS-specific, based on the post-hoc analysis in which we regarded the APLA-negative subgroup as non-APS controls. Lastly, SCFAs are linked to autoimmunity, for instance through effects on T helper cells and regulatory T cells. 25 Of the four measured SCFAs, we observed a small effect on fecal acetate levels only ( *p* = 0.04), but this was not statistically significant after correcting for multiple testing.

In conclusion, this study shows that the intestinal microbiota affect the biochemical disease activity in human APS through a yet unknown mechanism. Future studies should focus on unraveling this relation in pursuance of possible gut microbiome-directed treatment strategies.
