# Supplementary material for: An Intestinal Microbiome Intervention Affects Biochemical Disease Activity in Patients with Antiphospholipid Syndrome
Source: TH Open. 2024 Aug 5;8(3):e308–16. doi: 10.1055/s-0044-1788653 (PMC11300102; doi:10.1055/s-0044-1788653)
Supplement: Supplementary file 1 — Supplementary Material [file 10-1055-s-0044-1788653-s24040013.pdf]

Supplementary Materials and Methods

Biomarker Panel

Antiphospholipid Antibodies

In addition to criteria antiphospholipid antibodies, we also evaluated anti-cardiolipin immunoglobulin A (IgA) and anti-β2GPI IgA antibodies, because of their role in intestinal immunity, and we measured anti-phosphatidylserine/prothrombin IgM and IgG antibodies.

Lupus anticoagulant (LAC) testing was performed according to the International Society on Thrombosis and Haemostasis guidelines by both dilute Russell's viper venom time using STA-Staclot screen and confirm reagents (Stago, Asnières sur Seine, France), and silica clotting time HemosIL reagents (Werfen/Instrumentation Laboratories, Bedford, United States).[1] Patient plasma was mixed 1:1 with pooled normal plasma to exclude for coagulation factor deficiencies. Samples of four patients on direct oral anticoagulants were pretreated with DOAC-Remove20 (5-Diagnostics AG, Basel, Switzerland Ref. No. 5D-82410A). For all subjects, the LAC was included in the analysis as the numerical value of the normalized LA (nLA)-ratios, expressed as: (screen clotting time of patient/screen clotting time of pooled normal plasma)/(confirm clotting time of patient/confirm clotting time of pooled normal plasma). Quantitative values of anti-cardiolipin IgA, IgG, and IgM were measured with Cardiolipin IgA enzyme-linked immunosorbent assay (ELISA) kit (Abnova, Taoyuan City, Taiwan) or Cardiolipin IgM/IgG ELISA (IBL International IBL International GmbH, Hamburg, Germany). Anti-β2GPI IgA, IgG, and IgM were measured with β2GPI IgA ELISA kit (Creative Diagnostics, New York, United States) or IMTEC β2GPI antibodies IgG/IgM ELISA (Clindia Benelux BV, Almere, the Netherlands). Quantitative values of anti-phosphatidylserine/prothrombin IgG and IgM were measured with QUANTA Lite ELISA (Inova Diagnostics, San Diego, United States). Cut-off values for criteria antibodies (anti-cardiolipin IgG, IgM, and anti-β2GPI IgG, IgM) were determined based on a nonparametric 99th percentile of 120 reference individuals. Non-criteria antibody cut-off values (anti-phosphatidylserine/prothrombin IgG, IgM, anti-cardiolipin IgA, and anti-β2GPI IgA) were transferred from the manufacturer's recommendations.

Hemostatic Parameters

Platelet count was determined on a Sysmex XN-9000 System (Sysmex, Norderstedt Germany). Coagulation assays (prothrombin time and activated partial thromboplastin time) were performed on an automated coagulation analyzer (Behring Coagulation System, BCS) with reagents and protocols from the manufacturer (Siemens Healthineers, Marburg, Germany). D-dimer levels were determined with a particle-enhanced immunoturbidimetric assay (Innovance D-Dimer, Siemens Healthineers). Plasma von Willebrand factor antigen levels were measured by ELISA using antibodies from DAKO (Dakopatts P226 rabbit-anti-human von Willebrand antibodies).

Thrombin Generation and Activated Protein C Resistance

Thrombin generation in platelet-poor plasma (PPP) was determined by Calibrated Automated Thrombogram (Thrombinoscope BV, Maastricht, the Netherlands) as previously described[2] in the presence of phospholipids with two tissue factor concentrations: 1 and 5 pmol/L (Thrombinoscope BV, Maastricht, the Netherlands) using fluorogenic substrate in calcium buffer (FluCa, Thrombinoscope BV). Fluorescence was measured with Fluoroskan Ascent fluorometer (Thermo Labsystems OY, Helsinki, Finland). Thrombin generation curves were generated from the fluorescence signal using Thrombinoscope software (Thrombinoscope BV). The following parameters were included in the analysis: lag time, peak height, time to peak, velocity, and endogenous thrombin potential (ETP). To evaluate activated protein C (APC) resistance, thrombin generation was measured in presence of APC and 5 pmol/L tissue factor. ETP values measured in presence of APC were divided by ETP values in absence of APC to calculate APC sensitivity ratios (APC-sr). Sample APC-sr values were normalized by dividing by the APC-sr in normal pooled plasma. These normalized APC sensitivity ratios were included in the analysis.

Clot Lysis

Clot lysis was performed as previously described.[3] In short, 75 µL citrated plasma was mixed with recombinant human tissue factor (Innovin), $CaCl_2$, phospholipids, and tissue plasminogen activator. Optical density was measured at 405 nm to obtain a turbidity curve. Time from the midpoint between the clear and maximum turbidity (clot formation) to the midpoint between maximum turbidity and clear (clot lysis) was defined as clot lysis time. The clot lysis time was also measured in presence of carboxypeptidase inhibitor (CPI) to evaluate contribution of activation of thrombin-activatable fibrinolysis inhibitor. Clotting time, clot lysis time without CPI, and clot lysis time with CPI were included in the analysis.

Light Transmission Aggregometry

Subjects were either fasting or were allowed to have a light fat restricted meal before the first study visit. Light transmission aggregometry was performed within 3 hours after blood collection. Blood was drawn in citrate tubes using an open system. After 30 minutes, whole blood was centrifuged at 200G for 10 minutes at 19°C. To obtain PPP, the remaining blood was centrifuged at 4190G for 5 minutes at 19°C. The aggregometry was performed on the PAP8-E aggregometer (Sysmex, Etten-Leur, the Netherlands). First, the PPP was measured to set 100% light transmission. Then, platelet-rich

 © 2024. The Author(s).

plasma samples were incubated at 37°C and stirred at 1,000 rpm. Aggregation was induced by ADP at three concentrations: 2, 5, 10 µM (BIO/DATA Corporation/LS 101312). Initiation with TRAP 30 µM was used as positive control (Bachem H-8365). Aggregation curves were recorded for 10 minutes. For three concentrations of ADP, the following parameters were included in the analysis: primary slope, primary aggregation, maximal aggregation, and final aggregation.

Platelet Flow Cytometry

Platelet function flow cytometry was performed as previously described.[4] In short, citrullinated whole blood was added to a reagent mix consisting of HEPES buffered saline containing fluorescently labeled nanobodies against glycoprotein Ib α (GPIbα), p-selectin, fibrinogen, glycoprotein VI, integrin α2β1, or integrin αIIbβ3. Platelets were either unstimulated or stimulated with a receptor-specific agonist either ADP, CRP-xl, PAR-1 AP, PAR-4 AP, or U46619. Samples were incubated at 37°C for 10 minutes and then fixated for 15 minutes at room temperature. Immediately after fixation, samples were stored at 4°C and measured within 7 days on a BD FACSCanto flow cytometer. Platelets were identified using forward and side scatter and 10,000 platelets were measured for each parameter. Platelets identified based on forward and side scatter were then selected on GPIbα positivity or integrin αIIbβ3. Of the unstimulated integrin αIIbβ3-positive population percentages positive for glycoprotein VI and integrin α2β1 were included in the analysis. For the unstimulated and agonist stimulated platelets, the percentages positive for anti-P-selectin and anti-fibrinogen of the GPIbα-positive population were included in the analysis.

Complement and Inflammation Markers

C3a and C5a were measured in plasma that was kept on ice from blood draw until storage at −80°C. C3a and C5a plasma levels were determined with commercially available sandwich ELISA kits, according to the manufacturer's protocols (HK349–1115 and HK354–0913, respectively; Hycult Biotech, the Netherlands.)

CRP was determined in routine laboratory measurement on a Roche Cobas c702 (Roche Diagnostics, Basel, Switzerland). Tumor necrosis factor-α, interferon-γ, and interleukin-6 were determined with commercially available ELISA assay kits according to manufacturer's instructions (DTA00D, DIF50C, and D6050, respectively; R&D Systems, Inc., Minneapolis, United States).

Citrullinated Histone H3 was determined in citrate plasma with an commercially available ELISA kit (clone 11D3, 501620 Cayman Chemical, Ann Arbor, Michigan, United States) according to the instructions of the manufacturer.

Secondary Outcomes

Microbiome Sample and Data Processing

DNA was extracted from fecal material using a repeated bead beating protocol (method 5).[5] DNA was purified using Maxwell RSC Whole Blood DNA Kit. 16S rRNA gene amplicons were generated using a single-step polymerase chain reaction (PCR) protocol targeting the V3-V4 region.[6] PCR products were purified using Ampure XP beads and purified products were equimolar-pooled. The libraries were sequenced using a MiSeq platform using V3 chemistry with 2×251 cycles.

Forward and reverse reads were truncated to 240 and 210 bases respectively and merged using USEARCH.[7] Merged reads that did not pass the Illumina chastity filter, had an expected error rate higher than 2, or were shorter than 380 bases were filtered. Amplified sequence variants (ASVs) were inferred for each sample individually with a minimum abundance of four reads.[8] Unfiltered reads were than mapped against the collective ASV set to determine the abundances. Taxonomy was assigned using the RDP classifier[9] and SILVA[10] 16S ribosomal database V132. Contaminants were identified using decontam[11] software and subsequently, together with laboratory-specific known contaminants, removed from the dataset. Genera association scores with the first two components were calculated using vegans envfit function. Loadings for genera with the highest R-sqr were calculated using vegans wascores.

Lactulose Mannitol Test

We performed the lactulose/mannitol tests, a standard noninvasive method to measure intestinal permeability, at three time points (d0, d7, d42).[12] Subjects were instructed to fast starting from midnight of the day of examination. Subjects ingested a solution with 2 g of lactulose and 5 g of mannitol, two nonmetabolized sugars. Urine was collected for 3 hours and a urine sample was stored at −20°C until analysis. The ratio of both sugars excreted in urine reflects the intestinal barrier function as mannitol is normally absorbed, whereas lactulose is a large molecule that is only absorbed in case of decreased intestinal barrier function.

Measurement of Mannitol and Lactulose

Lactulose and mannitol were measured by gas chromatography with flame ionization detection, essentially as described in Jansen et al.[13] The limit of quantification for lactulose and mannitol is 0.05 mV/min (~40 µmol/L).

Lipocalin-2

Lipocalin-2 levels were measured in plasma using commercially available ELISA kit and following the manufacturer's protocol (Human Lipocalin-2/NGAL, R&D Systems, Minneapolis, Minnesota, United States).

Fecal Short-Chain Fatty Acids

Fecal short-chain fatty acid formate, acetate, propionate, and butyrate were measured with high-performance liquid chromatography with ultraviolet detection according to the method previously described by De Baere et al.[14] All measurements were corrected for wet and dry weight differences.

 © 2024. The Author(s).

Fecal Calprotectin

Fecal calprotectin levels were determined by automated immunoassay using EliA Calprotectin 2 on the Phadia 250 instrument (Thermo Fisher Scientific).

References

1 Devreese KMJ, de Groot PG, de Laat B, et al. Guidance from the Scientific and Standardization Committee for lupus anticoagulant/antiphospholipid antibodies of the International Society on Thrombosis and Haemostasis: Update of the guidelines for lupus anticoagulant detection and interpretation. J Thromb Haemost 2020;18(11):2828–2839

2 Hemker HC, Giesen P, Al Dieri R, et al. Calibrated automated thrombin generation measurement in clotting plasma. Pathophysiol Haemost Thromb 2003;33(01):4–15

3 Bakhtiari K, Kamphuisen PW, Mancuso ME, et al. Clot lysis phenotype and response to recombinant factor VIIa in plasma of haemophilia A inhibitor patients. Br J Haematol 2013;162(06):827–835

4 van Asten I, Schutgens REG, Baaij M, et al. Validation of flow cytometric analysis of platelet function in patients with a suspected platelet function defect. J Thromb Haemost 2018;16(04):689–698

5 Costea PI, Zeller G, Sunagawa S, et al. Towards standards for human fecal sample processing in metagenomic studies. Nat Biotechnol 2017;35(11):1069–1076

6 Kozich JJ, Westcott SL, Baxter NT, Highlander SK, Schloss PD. Development of a dual-index sequencing strategy and curation pipeline for analyzing amplicon sequence data on the MiSeq Illumina sequencing platform. Appl Environ Microbiol 2013;79(17):5112–5120

7 Edgar RC. Search and clustering orders of magnitude faster than BLAST. Bioinformatics 2010;26(19):2460–2461

8 Edgar RC. UNOISE2: improved error-correction for Illumina 16S and ITS amplicon sequencing. BioRxiv 2016:081257. https://doi.org/10.1101/081257

9 Wang Q, Garrity GM, Tiedje JM, Cole JR. Naive Bayesian classifier for rapid assignment of rRNA sequences into the new bacterial taxonomy. Appl Environ Microbiol 2007;73(16):5261–5267

10 Quast C, Pruesse E, Yilmaz P, et al. The SILVA ribosomal RNA gene database project: improved data processing and web-based tools. Nucleic Acids Res 2013;41(Database issue):D590–D596

11 Davis NM, Proctor DM, Holmes SP, Relman DA, Callahan BJ. Simple statistical identification and removal of contaminant sequences in marker-gene and metagenomics data. Microbiome 2018;6(01):226

12 Sequeira IR, Lentle RG, Kruger MC, Hurst RD. Standardising the lactulose mannitol test of gut permeability to minimise error and promote comparability. PLoS One 2014;9(06):e99256

13 Jansen G, Muskiet FAJ, Schierbeek H, Berger R, van der Slik W. Capillary gas chromatographic profiling of urinary, plasma and erythrocyte sugars and polyols as their trimethylsilyl derivatives, preceded by a simple and rapid prepurification method. Clin Chim Acta 1986;157(03):277–293

14 De Baere S, Eeckhaut V, Steppe M, et al. Development of a HPLC-UV method for the quantitative determination of four short-chain fatty acids and lactic acid produced by intestinal bacteria during in vitro fermentation. J Pharm Biomed Anal 2013;80:107–115

 © 2024. The Author(s).
